# Sterol 27-Hydroxylase Deficiency as a Cause of Neonatal Cholestasis: Report of 2 Cases and Review of the Literature

**DOI:** 10.3389/fped.2020.616582

**Published:** 2021-01-13

**Authors:** Patryk Lipiński, Maja Klaudel-Dreszler, Elzbieta Ciara, Dorota Jurkiewicz, Rafał Płoski, Joanna Cielecka-Kuszyk, Piotr Socha, Irena Jankowska

**Affiliations:** ^1^Department of Pediatrics, Nutrition and Metabolic Diseases, The Children's Memorial Health Institute, Warsaw, Poland; ^2^Department of Gastroenterology, Hepatology, Feeding Disorders and Pediatrics, The Children's Memorial Health Institute, Warsaw, Poland; ^3^Department of Medical Genetics, The Children's Memorial Health Institute, Warsaw, Poland; ^4^Department of Medical Genetics, Medical University of Warsaw, Warsaw, Poland; ^5^Department of Pathology, The Children's Memorial Health Institute, Warsaw, Poland

**Keywords:** neonatal cholestasis, sterol 27-hydroxylase (CYP27A1), next-generation sequencing, chenodeoxycholic acid (CDCA), liver transplantation

## Abstract

**Introduction:** Inborn errors of primary bile acid (BA) synthesis are rare autosomal recessive disorders responsible for 1–2% of cases of neonatal cholestasis. Among them, cerebrotendinous xanthomatosis (CTX) is caused by mutations in the *CYP27A1* gene resulting in the impairment of sterol 27-hydroxylase enzyme activity.

**Patients and Methods:** Here we present the study on two siblings with neonatal cholestasis diagnosed with sterol 27-hydroxylase deficiency. The clinical, biochemical, histological, and molecular presentation at the time of diagnosis and detailed follow-up were described. An extensive overview of the literature regarding patients with sterol 27-hydroxylase deficiency presenting with neonatal cholestasis was also provided.

**Results:** Patient 1 presented with cholestatic jaundice since 10 weeks of age and developed the end-stage liver disease requiring liver transplantation at 8 months of age but finally succumbed 3 years post-transplantation due to autoimmune hemolytic anemia and multiorgan failure development. Next-generation sequencing performed *post mortem*, revealed him to be homozygous for the known pathogenic splicing variant c.1184+1G>A in the *CYP27A1* gene. Patient 2 (sibling) presented with cholestatic jaundice since the first day of life. Sanger sequencing of *CYP27A1* revealed the same results. Chenodeoxycholic acid treatment was introduced just after diagnosis, at 4 months of age. Fourteen patients with sterol 27-hydroxylase deficiency presenting with neonatal cholestasis were reported in the literature, in most of them presenting as a self-limiting disease.

**Conclusions:** An early recognition and treatment initiation in CTX is essential.

## Introduction

Inborn errors of primary bile acid (BA) synthesis are rare autosomal recessive disorders responsible for 1–2% of cases of neonatal cholestasis (1–3). The absence of pruritus, normal serum gamma-glutamyltranspeptidase (GGT) activity, and normal or low total serum BA concentration constitute the most characteristic features while the specific diagnosis is based on the mass spectrometry (MS) analysis of urinary BA confirmed by gene sequencing (1–4).

Cerebrotendinous xanthomatosis (CTX; # 213700) is caused by mutations in the *CYP27A1* gene resulting in the impairment of sterol 27-hydroxylase enzyme (EC 1.14.15.15) activity with subsequent diminished cholic acid (CA) formation and no production of chenodeoxycholic acid (CDCA) (5–7). An increased deposition of cholesterol and cholestanol in tissues throughout the body, including the brain (white matter), lens, and tendons is observed (5). Clinical hallmarks include premature bilateral cataracts, intractable diarrhea, tendon xanthomas, and progressive neurologic dysfunction (5–7). Early diagnosis remains crucial regarding that early and long-term treatment of CDCA improves the neurological outcome and even could reverse the progression of the disease (8–10).

Here we present the study on two siblings with neonatal cholestasis diagnosed with sterol 27-hydroxylase deficiency based on molecular analysis, **aiming** to highlight the manifestation of disease as neonatal cholestasis, emphasize the diagnostic difficulties with depicting the role of genetic studies, and also report the favorable outcome of an oral CDCA therapy. A detailed overview of the literature regarding sterol 27-hydroxylase deficiency presenting with neonatal cholestasis was also provided.

## Patients and Methods

The presentation at the time of diagnosis and detailed follow-up were described. An informed and written consent was obtained from the parents of patients. Ethical approval was obtained from the Children's Memorial Health Institute Bioethical Committee, Warsaw, Poland.

A retrospective chart review of patients' medical records concerning the biochemical (hemoglobin, platelets, serum transaminases, total and direct serum bilirubin, gamma-glutamyl transpeptidase, internal normalized ratio, serum bile acids), histopathological (microscopic examination of liver biopsy specimens) and molecular (*CYP27A1* gene mutations) were collected.

The liver biopsy specimens, between 1.0 and 1.5 cm in length, were fixed in 4% formalin and stained by hematoxillin and eosin, PAS method (periodic acid + Schiff reagent) and PAS method after diastase digestion, Azan method and reticulin impregnation. To assess the histological activity of microscopical changes, the following categories of lesions were considered retrospectively: presence of inflammatory infiltrates in the portal spaces and lobules with or without piecemeal necrosis, degree of fibrosis, lobular disarray, rosette formations, proliferation of bile ducts and ductules with or without ductitis, lobular necrosis, hepatocyte giant cell transformation, steatosis and degenerative changes in the hepatocytes, canalicular bile plugs, cholestasis in the hepatocytes and in bile ducts, extramedullary hematopoesis.

Genomic DNA was extracted from peripheral blood samples of the patients. Next-generation sequencing (NGS) of targeted gene panel, created by The Children's Memorial Health Institute's for the simultaneous sequencing of 1,000 clinically relevant genes including 53 items related to cholestatic liver disorders was used. A detailed protocol have been published recently (11).

The nomenclature of molecular variants follows the Human Genome Variation Society guidelines (HGVS, http://varnomen.hgvs.org/) using human cDNA sequence of appropriate genes followed the Human Gene Mutation Database (HGMD, http://www.hgmd.cf.ac.uk).

The database PubMed (MEDLINE) was searched for relevant studies on September 30, 2020. The following keyword-based strategy was used: (“cerebrotendinous xanthomatosis” OR “CTX” OR “sterol 27-hydroxylase deficiency”) AND (“neonatal cholestasis”). All studies, letters, and abstracts that contained sufficient data were included. Available data regarding the first presentation, age at referral, biochemical and histological features, treatment and outcome, were extracted.

### Patients' Presentation

#### Patient 1

**Figure 1:**
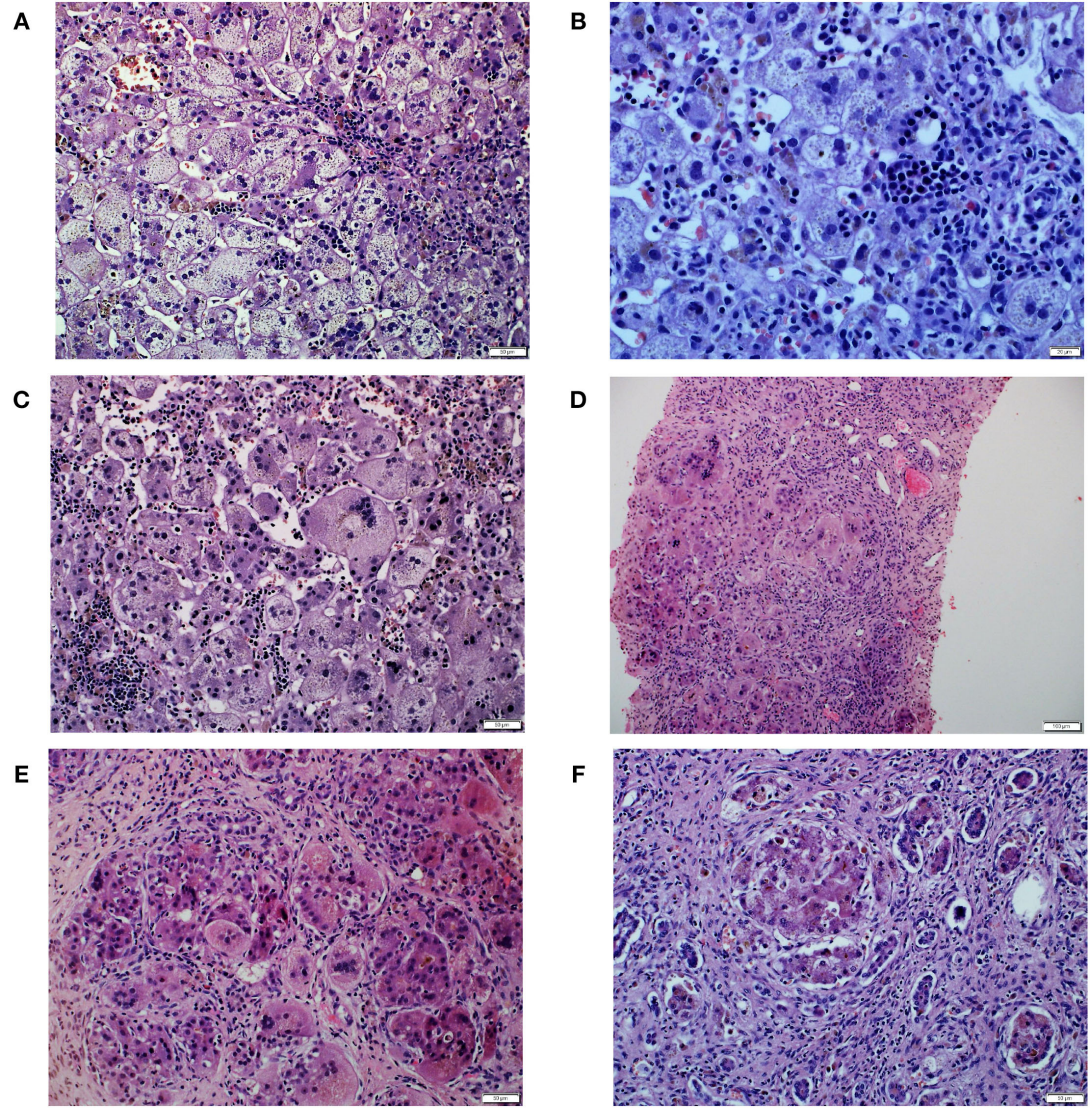
Histopathological liver features in Patient 1. **(A)** Diffuse inflammatory infiltrates composed of lymphoid cells, in the portal tracts. **(B)** Abundant foci of extramedullary heamatopoiesis in the sinusoids. **(C)** Multinucleated giant hepatocytes and hepatic rosettes with bilirubinostasis. **(D)** Multinucleated giant cells were inside cirrhotic nodules. **(E)** Intracellular cholestasis with non-specific inflammatory infiltrates composed of neutrophils and lymphocytes in the fibrous septa. **(F)** Late cirrhosis with only small remnants of liver cells surrounded by fibrous tissue.

The patient was the first child of non-consanguineous Polish parents born from an uneventful pregnancy at 39 weeks of gestation with a birth mass of 3,000 g. At the age of 10 weeks, he was referred to our hospital due to presence of jaundice. No history of hypo-/acholic stools was observed. Cholestasis with normal serum GGT accompanied by elevated serum transaminases and coagulopathy was diagnosed (Table 1). Normal liver and spleen volume were observed. HBV, HCV, CMV, EBV, HIV, and *Toxoplasma gondii* infections were excluded serologically; alpha-1-antitrypsin deficiency, cystic fibrosis, galactosemia were excluded as well. The liver biopsy performed at the age of 77 days revealed diffuse inflammatory infiltrates composed of lymphoid cells, situated in the portal tracts (Figure 1A). Abundant foci of extramedullary heamatopoiesis were found intraacinar in the sinusoids (Figure 1B). Multinucleated giant hepatocytes and hepatic rosettes with bilirubinostasis were found in all acini (Figure 1C). The patient was discharged home on ursodeoxycholic acid (UDCA) as well as fat-soluble vitamins treatment.

**Table 1:** Results of laboratory analyses of Patient 1.

**Parameter and reference values**	**Age**							
	**10 weeks**	**4 months**		**5 months**		**9 months**	**2 years**	**3.5 years**
Hemoglobin [g/dl]	13.5	13.0		12.5		12.5	6.3	6.5
Platelets [150–450 K/ul]	400	197		86		381	291	304
Total serum bilirubin [<1.00 mg/dl]	9.2	18.1		28.6		1.5	0.56	0.33
Direct serum bilirubin [<0.2 mg/dl]	7.9	14.4	PEBD	25.2	LTx	0.65	0.37	0.31
AST [<60 U/l]	1137	2139		709		117	62	50
ALT [<60 U/l]	704	1935		411		76	40	46
GGTP [<200 U/l]	32	21		23		36	169	280
INR [0.9–1.3]	Not	1.25		1.9		0.81	1.09	1.1
Albumin [3.8–5.4 g/dl]	3.7	3.9		2.5		4.3	3.84	4.1
Serum bile acids [<10.0 umol/l]	63.1	220		278		<10.0	64.2	n.a.

At the age of 4 months he was hospitalized in the Hepatology Outpatient Clinic. Notably, raising parameters of cholestasis with high serum BA concentration as well as still highly elevated serum transaminases were noted (Table 1). The decision about partial external biliary diversion (PEBD) procedure was commenced. The liver biopsy done at the moment of PEBD revealed the presence of severe fibrosis with features of cirrhotic transformation. Multinucleated giant cells were seen inside cirrhotic nodules (Figure 1D). Intracellular cholestasis was more prominent as compared to the first biopsy and non-specific inflammatory infiltrates composed of neutrophils and lymphocytes were found in the fibrous septa (Figure 1E).

During control visit in the Hepatology Outpatient Clinic, 4 weeks after PEBD, the child presented with jaundice, huge ascites, and severe itching. Laboratory results revealed raising parameters of cholestasis with high serum BA concentration as well as coagulopathy and hypoalbuminemia (Table 1). The decision about qualification for liver transplantation (LTx) procedure was made.

The patient underwent AB0-compatible cadaveric liver transplantation (LTx) at 8 months of age. The examination of the explanted liver revealed features of late cirrhosis with only small remnants of liver cells surrounded by fibrous tissue (Figure 1F). An early post-LTx outcome was complicated with an acute rejection episode (1 month after LTx) and biliary complications (anastomotic strictures diagnosed 3 months after LTx). At the age of 2 years (1 year and 2 months after LTx) he developed a hemolytic anemia related to production of cold agglutinins (Table 1). Viral serologies significant for cold agglutinin syndrome were negative. No clinical evidence of post-transplant lymphoproliferative disorder was observed. Favorable resolution of the hemolytic anemia occurred following typical treatment with glucocorticosteroids.

The recurrence of hemolytic anemia refractory to steroids, plasmapheresis, and rituximab was observed at 3.5 years of age. The patient had finally died due to multiorgan failure development.

#### Patient 2

The patient was the younger sister of Patient 1, born from an uneventful second pregnancy at 40 weeks of gestation with a birth mass of 2,950 g. At the age of 9 days of life, she was referred to our hospital due to presence of jaundice observed since the first day of life. No history of hypo-/acholic stools was observed. Cholestasis with normal serum GGT accompanied by slightly elevated serum transaminases was diagnosed (Table 2). Normal liver and spleen volume were observed. The patient was discharged home on UDCA as well as fat-soluble vitamins treatment.

**Table 2:** Results of laboratory analyses of Patient 2.

**Parameter and reference values**	**Age**				
	**9 days of life**	**2 months**		**6 months**	**1 y 4 months**
Platelets [150–450 K/ul]	340	640		320	410
Total serum bilirubin [<1.00 mg/dl]	8.1	5.5	CDCA treatment since 4 months	0.74	0.15
Direct serum bilirubin [<0.2 mg/dl]	3.6	4.7		0.18	0.09
AST [<60 U/l]	85	369		95	41
ALT [<60 U/l]	30	276		134	22
GGTP [<200 U/l]	95	77		28	11
INR [0.9–1.3]	0.94	1.04		0.96	0.92
Albumin [3.8–5.4 g/dl]	3.7	4.3		4.5	4.9
Serum bile acids [<10.0 umol/l]	56.3	131		30.4	6.1

At the age of 2 months she was hospitalized in the Hepatology Outpatient Clinic. Notably, raising parameters of cholestasis with high serum BA concentration as well as serum transaminases levels were noted (Table 2).

In the meanwhile, the results of next-generation sequencing study of her brother became available—the patient was found to be homozygous for the known pathogenic splicing variant c.1184+1G>A in the *CYP27A1* gene (NM_000784.3). The parents were carriers of this variant. The Sanger sequencing of *CYP27A1* in his sister revealed the same results; the diagnosis of CTX was established. The decision about chenodeoxycholic acid (CDCA) treatment was made and CDCA was finally introduced at the age of 4 months at a dosage of 5 mg/kg/day. After 1 year of treatment with CDCA maintained on a dosage of 5 mg/kg/day, the patient was in a good clinical condition presenting with normal results of liver function tests (Table 2). The therapy was well-tolerated by the patient.

### Literature Review

Fourteen patients with sterol 27-hydroxylase deficiency presenting with neonatal cholestasis were reported, see Table 3 (11–19). To be noted that a detailed clinical presentation was not available in some of the reported cases (12, 14, 17, 19).

**Table 3:** Review on reported patients with sterole 27-hydroxylase deficiency presenting with neonatal cholestasis.

**Patient**	**First presentation**	**Age at referral**	**Biochemical and histological features**	**Treatment and outcome**	**References**
1	Jaundice since 7 days of life	9 weeks	AST 575 IU/L, GGT 38 I/U, albumin 38 g/L; INR 1.2; pruritus since 0.3 y Liver biopsy: a non-specific hepatitis with giant cells and a few areas of focal bridging necrosis, marked inflammatory cell infiltrate and very marked cholestasis.	CDCA (10 mg/kg) from 0.47 y; at 0.49 y the bile acid therapy changed to CDCA (5 mg/kg) and CA (5 mg/kg); liver biopsy at 0.52 y—cirrhosis with cholestasis and active (although reduced) portal inflammation Follow-up: Treatment with chenodeoxycholic acid and cholic acid led to a resolution of the hepatitis and at the of 12y he was asymptomatic with normal liver function tests Their first child died at 13 mo, having been jaundiced all his life	(12, 13)
2	Apart from neonatal unconjugated hyperbilirubinemia treated with phototherapy, the immediate postnatal period was seen as normal	6 weeks	Liver biopsy: profound giant cell transformation with an extremely high number of nuclei per hepatocyte, very little lymphocytic infiltration and moderate bile duct proliferation	Ongoing CMV infection Death at 4 mo	(14)
3	Jaundice shortly after birth	3 mo	AST 275 IU/L; GGT 95 I/U Liver biopsy: chronic active hepatitis with piecemeal necrosis and moderate fibrosis	Outcome: jaundice resolved spontaneously by 5 mo of age and his liver function normalized by 8 mo Started on CA (15 mg/kg) at age 14 mo Follow-up: 8 y—clinically normal without any xanthomas or cataracts	(15)
4	Feeding difficulties, lethargy, temperature instability, convulsions. No jaundice present at this time	8 days of life	Coagulopathy (prolonged prothrombin time). Elevated plasma cholestanol, low CDCA, and CA	Treatment: CDCA at a dosage of 15 mg/kg/day stopped after 6 weeks due to hepatoxicity. CDCA restarted at a dosage of 5 mg/kg/day during 2.5 years of treatment with good results	(16)
5	Jaundice from age 4 days, gradually fading but recurrent at age 2 mo	4 mo	In all 3 patients (5, 6, and 8) who underwent liver biopsy, light microscopy found intralobular cholestasis (hepatocellular and canalicular), and giant-cell transformation of hepatocytes, with some necrotic hepatocytes. Portal tract fibrosis and ductular reaction were uniformly present In the explanted liver of patient 10, liver cirrhosis with moderate activity accompanied changes like those seen in patients 5, 6, and 8, were observed.	Treatment: UDCA, ganciclovir (CMV infection), prednisone Death at 8 mo	(17)
6	Jaundice from age 3 days, worsening throughout first postnatal month	4 mo		Treatment: ganciclovir (CMV infection), methylprednisolone Death at 7 mo	
7	Jaundice from age 10 days with acholic stools	6 mo		Treatment: UDCA, ganciclovir (CMV infection) Death at 9 mo	
8	Jaundice from age 2 days, with acholic stools	3 mo		Treatment: None Death at 5 mo	
9	Jaundice from age 7 days, worsening from age 1 mo, with acholic stools	2 mo		Treatment: UDCA Alive at 17 mo	
10	Jaundice after born, gradual fading but worsening again from age 3 mo	3 mo		Treatment: UDCA Alive at 15 mo	
11	Jaundice from age 5 days, worsening from age 1 mo with dark urine; behavior suggesting pruritus from age 5 mo	7 mo		Treatment: UDCA, ganciclovir (CMV infection), LTx at 8 mo Alive at 13 mo	
12	Jaundice from age 3 days with acholic stools	3 mo		Treatment: UDCA, methylprednisolone Alive at 12 mo	
13	Jaundice from 2 mo, developmental delay	Not known	Liver biopsy (8 mo): liver cirrhosis	Treatment: CDCA, LTx at 8 mo Follow-up: normal liver function 1 year after LTx	(11)
14	Jaundice from 2 mo	2 mo	AST 473 IU/L, ALT 530 IU/L, GGT normal Liver biopsy: idiopathic hepatocellular cholestasis such as pronounced lobular disarray, giant-cells, bile pigment within hepatocytes, with slightly enlarged portal tracts in the absence of bile duct proliferation	UDCA started at 3 mo. Normalization of bilirubin and transaminases, respectively, at 6 and 8 months. UDCA was stopped and CDCA (10–15 mg/kg) was started at 8 mo age Follow-up: 13 y. Normal liver function tests, no xanthomas, normal cognitive development	(18)

## Discussion

The paper presents a detailed clinical, biochemical, and histological phenotype of two patients (siblings) diagnosed with sterol 27-hydroxylase deficiency (cerebrotendinous xanthomatosis, CTX) manifested with neonatal cholestatic jaundice. The first patient, in fact, without adequate therapy progressed to liver failure and underwent LTx but finally died. CTX diagnosis was established *post mortem*. The second patient (sibling) was diagnosed with CTX at 4 months of age by next-generation sequencing (NGS) study. This fact raises the usefulness of NGS techniques in diagnostic approach to neonatal cholestasis.

The diagnosis of inborn errors of primary BA synthesis remains challenging, especially in the differential diagnosis of neonatal/infantile cholestasis. The absence of pruritus, normal serum GGT activity, and normal or low total serum BA concentration are the most characteristic features of inborn errors of BA synthesis (1, 2). In some patients (like *Patient 1* and some reported patients) despite of lack of itching at first, a severe itching was observed in the course of disease. That, based on the clinical and biochemical features a diagnosis of progressive familial intrahepatic cholestasis (PFIC) could be established. PFIC patients generally present in the first few months of life with cholestatic jaundice and pruritus, high serum BA and transaminases, normal serum GGT levels (beyond PFIC type 3). Liver histology shows usually a marked intracellular cholestasis and an obvious giant-cell transformation. In our patient, the histopathological liver study was also miscellaneous (20). The massive gigantocellular transformation of hepatocytes as well as extramedullary hematopoiesis were not characteristic for PFIC. However, the giant-cell transformation of hepatocytes was the most common histopathological finding reported in patients with cholestatic liver disease in the course of CTX (Table 3). Thus, we recommend to consider CTX in a patient presenting with neonatal cholestasis, especially in the presence of giant cell hepatitis.

In the case of prolonged intrahepatic cholestasis with low levels of GGT, without other explanations, the analysis of bile alcohols by mass spectrometry is indicated. BA profiles of plasma and urine are useful in CTX diagnosis but depend on methodology, sample type and preparation, and experience of the laboratory performing the test. In our country these techniques are not routinely available (2). The introduction of NGS allowed us to diagnose CTX and other disorders which have not been recognized yet (21). Molecular biology constitutes an useful diagnostic tool, especially when mass spectrometry data may be inconclusive, such as during the early neonatal period, or if the patient had been treated with bile acid before analysis (13, 17).

There is an existing literature including diagnosis and some outcomes of infants with neonatal cholestasis with CTX. In 1995, Clayton et al. described the first infant with giant cell hepatitis in the course of CTX in whom the treatment with CDCA and CA led to a resolution of the hepatitis at the age of 12 years (12). The retrospective analysis of the past medical records of 50 Dutch patients with CTX, authored by Clayton et al., revealed that neonatal liver disease defined as prolonged jaundice with raised serum transaminases occurred much more often in CTX than in the general population (13). Up to now, neonatal cholestatic jaundice in the course of CTX has been reported in 14 patients, in most of them presenting as a self-limiting disease (11–19). However, a fatal neonatal cholestasis has been reported in 5 out of 12 patients (Table 3). In the largest cohort of 8 infants with CTX presenting with severe cholestasis, reported by Gong et al., 4 died from liver failure at the age of 8, 7, 9, and 5 months, respectively; in 3 other patients their jaundice resolved spontaneously. The authors proposed that CTX manifested as neonatal cholestasis could have a relatively adverse prognosis. One child had undergone LTx at 8 months of age; at the last follow-up (17 months) was presenting with a good outcome (17). Shen et al. reported the second child in the literature who underwent LTx (11). This patient did not require longer CDCA supplement therapy; LTx not only restored liver function but also corrected cholestanol metabolism. LTx could correct the metabolic defect but it is not the advisable therapeutic strategy for CTX, since there is an effective and safe therapy for this condition.

Early diagnosis remains crucial regarding that early and long-term treatment of CDCA improves the neurological outcome and even could reverse the progression of the disease (8–10). Our patient was started to treat with CDCA at 4 months at a dosage of 5 mg/kg/day. After 1 year of treatment with CDCA maintained on a dosage of 5 mg/kg/day, the patient was in a good clinical condition presenting with normal results of liver function tests. CDCA has been reported as hepatotoxic in one infant who started CDCA treatment at a dosage of 15 mg/kg/day but also as safe and effective in other pediatric patients (16). Degrassi et al. have recently reported a patient who started to be treated at 8 months of age with the initial dose of 10 mg/kg/day, increased until 15 mg/kg/day. The patient has been followed for 13 years and the therapy was well-tolerated (18).

The main limitation of our study is no biochemical data to confirm diagnosis of CTX and also follow-up of these patients. In our country it is not possible to perform the analysis of bile alcohols with tandem-mass spectrometry. The observation that CTX can present with neonatal cholestasis is also not novel thus an extensive review of the literature on pediatric cases of neonatal CTX cholestasis was provided, which may be helpful to stress the importance of an early diagnosis of this condition.

## Conclusions

1. We recommend to consider CTX in a patient presenting with neonatal cholestasis of an unknown origin, especially in the presence of giant cell hepatitis.
2. An early recognition and treatment initiation in CTX is essential.
3. Panel-based next-generation sequencing could be a useful screening tool in patients with neonatal cholestais of an unknown origin, especially when other biochemical methods such us analysis of bile alcohols by mass spektrometry are not available.

## Data Availability Statement

The original contributions presented in the study are included in the article/supplementary material, further inquiries can be directed to the corresponding author/s.

## Ethics Statement

The studies involving human participants were reviewed and approved by ethics committee of The Children's Memorial Health Institute in Warsaw. Written informed consent to participate in this study was provided by the participants' legal guardian/next of kin. Written informed consent was obtained from the minor(s)' legal guardian/next of kin for the publication of any potentially identifiable images or data included in this article.

## Author Contributions

PL and IJ: project administration. IJ: supervision. PL, MK-D, EC, DJ, RP, JC-K, PS, and IJ: investigation and writing – review and editing. PL: writing – original draft. All authors contributed to the article and approved the submitted version.

## Conflict of Interest

The authors declare that the research was conducted in the absence of any commercial or financial relationships that could be construed as a potential conflict of interest.

## References

[1] Sundaram SS Bove KE Lovell MA Sokol RJ. Mechanisms of disease: inborn errors of bile acid synthesis. Nat Clin Pract Gastroenterol Hepatol. (2008) 5:456–68. 10.1038/ncpgasthep1179 18577977 PMC3888787

[2] Heubi JE Setchell KDR Bove KE. Inborn errors of bile acid metabolism. Clin Liver Dis. (2018) 22:671–87. 10.1016/j.cld.2018.06.006 30266156

[3] Fawaz R Baumann U Ekong U Fischler B Hadzic N Mack CL. Guideline for the evaluation of cholestatic jaundice in infants: joint recommendations of the North American Society for Pediatric Gastroenterology, Hepatology, and Nutrition and the European Society for Pediatric Gastroenterology, Hepatology, and Nutrition. J Pediatr Gastroenterol Nutr. (2017) 64:154–68. 10.1097/MPG.0000000000001334 27429428

[4] Gonzales E Matarazzo L Franchi-Abella S Dabadie A Cohen J Habes D. Cholic acid for primary bile acid synthesis defects: a life-saving therapy allowing a favorable outcome in adulthood. Orphanet J Rare Dis. (2018) 13:190. 10.1186/s13023-018-0920-5 30373615 PMC6206929

[5] Nie S Chen G Cao X Zhang Y. Cerebrotendinous xanthomatosis: a comprehensive review of pathogenesis, clinical manifestations, diagnosis, and management. Orphanet J Rare Dis. (2014) 9:179. 10.1186/s13023-014-0179-4 25424010 PMC4264335

[6] Salen G Steiner RD. Epidemiology, diagnosis, and treatment of cerebrotendinous xanthomatosis(CTX). J Inherit Metab Dis. (2017) 40:771–81. 10.1007/s10545-017-0093-8 28980151

[7] Duell PB Salen G Eichler FS DeBarber AE Connor SL Casaday L. Diagnosis, treatment, and clinical outcomes in 43 cases with cerebrotendinous xanthomatosis. J Clin Lipidol. (2018) 12:1169–78. 10.1016/j.jacl.2018.06.008 30017468

[8] Mandia D Chaussenot A Besson G Lamari F Castelnovo G Curot J. Cholic acid as a treatment for cerebrotendinous xanthomatosis in adults. J Neurol. (2019) 266:2043–50. 10.1007/s00415-019-09377-y 31115677

[9] Stelten BML Huidekoper HH van de Warrenburg BPC Brilstra EH Hollak CEM Haak HR. Long-term treatment effect in cerebrotendinous xanthomatosis depends on age at treatment start. Neurology. (2019) 92:e83–95. 10.1212/WNL.0000000000006731 30530799

[10] Wong JC Walsh K Hayden D Eichler FS. Natural history of neurological abnormalities in cerebrotendinousxanthomatosis. J Inherit Metab Dis. (2018) 41:647–56. 10.1007/s10545-018-0152-9 29484516

[11] Shen CH Wang ZX. Liver transplantation due to cerebrotendinous xanthomatosis end-stage liver disease. World J Pediatr. (2018) 14:414–15. 10.1007/s12519-018-0151-9 29796951

[12] Clayton PT Casteels M Mieli-Vergani G Lawson AM. Familial giant cell hepatitis with low bile acid concentrations and increased urinary excretion of specific bile alcohols: a new inborn error of bile acid synthesis? Pediatr Res. (1995) 37:424–31. 10.1203/00006450-199504000-00007 7596681

[13] Clayton PT Verrips A Sistermans E Mann A Mieli-Vergani G Wevers R. Mutations in the sterol 27-hydroxylase gene (CYP27A) cause hepatitis of infancy as well as cerebrotendinous xanthomatosis. J Inherit Metab Dis. (2002) 25:501–13. 10.1023/A:1021211520034 12555943

[14] von Bahr S Björkhem I Van't Hooft F Alvelius G Nemeth A Sjövall J. Sterol 27-hydroxylase gene associated with fatal cholestasis in infancy. J Pediatr Gastroenterol Nutr. (2005) 40:481–6. 10.1097/01.MPG.0000150419.23031.2A 15795599

[15] Pierre G Setchell K Blyth J Preece MA Chakrapani A McKiernan P. Prospective treatment of cerebrotendinous xanthomatosis with cholic acid therapy. J Inherit Metab Dis. (2008) 31:241–45. 10.1007/s10545-008-0815-z 19125350

[16] Huidekoper HH Vaz FM Verrips A Bosch AM. Hepatotoxicity due to chenodeoxycholic acid supplementation in an infant with cerebrotendinous xanthomatosis: implications for treatment. Eur J Pediatr. (2016) 175:143–6. 10.1007/s00431-015-2584-7 26156051 PMC4709371

[17] Gong JY Setchell KDR Zhao J Zhang W Wolfe B Lu Y. Severe neonatal cholestasis in cerebrotendinous xanthomatosis: genetics, immunostaining, mass spectrometry. J Pediatr Gastroenterol Nutr. (2017) 65:561–8. 10.1097/MPG.0000000000001730 28937538

[18] Degrassi I Amoruso C Giordano G Del Puppo M Mignarri A Dotti MT. Case report: early treatment with chenodeoxycholic acid in cerebrotendinous xanthomatosis presenting as neonatal cholestasis. Front Pediatr. (2020) 8:382. 10.3389/fped.2020.00382 32766184 PMC7381104

[19] Setchell KDR O'Connell N Russell DW A unique case of cerebrotendinous xanthomatosis presenting in infancy with cholestatic liver disease further highlights bile acid synthetic defects as an important category of metabolic liver disease (abstract). In: Van Berge Henegouwen GP Keppler D Leuschner U Paumgartner G Stiehl A, editors. Falk Symposium 120. XVI International Bile Acid Meeting. Biology of Bile Acids in Health and Disease. Den Haag (The Netherlands). Dordrecht: Kluwer (2001). p. 13–4.

[20] Bull LN Thompson RJ. Progressive familial intrahepatic cholestasis. Clin Liver Dis. (2018) 22:657–69. 10.1016/j.cld.2018.06.003 30266155

[21] Lipiński P Ciara E Jurkiewicz D Pollak A Wypchło M Płoski R. Targeted next-generation sequencing in diagnostic approach to monogenic cholestatic liver disorders-single-center experience. Front Pediatr. (2020) 8:414. 10.3389/fped.2020.00414 32793533 PMC7393978

